# Supplementary material for: Network Analysis of Neurobehavioral Symptom Patterns in an International Sample of Spanish-Speakers with a History of COVID-19 and Controls
Source: Int J Environ Res Public Health. 2022 Dec 23;20(1):183. doi: 10.3390/ijerph20010183 (PMC9819652; doi:10.3390/ijerph20010183)
Supplement: Supplementary file 1 [file ijerph-20-00183-s001.zip › ijerph-2043465-supplementary.pdf]

Supplemental Document 1

Table S1

Descriptive Statistics from the COVID- Group.

Variable	*M*	*SD*	Min	Max	Skew	Kurtosis	*SE*
Annoyed	1.88	0.88	1	4	0.70	-0.37	0.04
Appetite	1.17	0.48	1	4	3.18	11.11	0.02
Balance	1.38	0.67	1	4	1.89	3.47	0.03
Clumsy	1.43	0.68	1	4	1.55	1.96	0.03
Concentrate	1.64	0.86	1	4	1.20	0.54	0.04
Decide	1.88	0.86	1	4	0.69	-0.30	0.04
Dizzy	1.58	0.75	1	4	1.18	0.80	0.04
Fatigue	1.6	0.88	1	4	1.31	0.67	0.04
Forget	1.86	0.97	1	4	0.85	-0.37	0.05
Frustrated	1.77	0.88	1	4	0.95	0.07	0.04
Head	1.52	0.78	1	4	1.44	1.36	0.04
Hearing	1.65	0.85	1	4	1.15	0.44	0.04
Light	1.71	0.85	1	4	0.95	-0.07	0.04
Nausea	2.15	0.91	1	4	0.28	-0.85	0.04
Noise	1.35	0.66	1	4	2.02	3.80	0.03
Numb	1.51	0.79	1	4	1.50	1.51	0.04
Organize	1.63	0.81	1	4	1.13	0.52	0.04
Sad	1.83	0.91	1	4	0.87	-0.16	0.04
Sleep	2	0.93	1	4	0.55	-0.64	0.04
Taste	1.67	0.82	1	4	1.10	0.53	0.04
Tense	2.01	0.93	1	4	0.51	-0.72	0.04
Vision	1.37	0.62	1	4	1.65	2.57	0.03

Table S2

Descriptive Statistics from the COVID+ Group.

Variable	*M*	*SD*	Min	Max	Skew	Kurtosis
Annoyed	650	1.87	0.94	0.98	0.47	0.04
Appetite	650	1.78	1.02	1.17	0.55	0.04
Balance	650	1.46	0.75	1.69	2.61	0.03
Clumsy	650	1.5	0.77	1.67	2.87	0.03
Concentrate	650	1.66	0.90	1.21	0.78	0.04
Decide	650	2.07	1.03	0.67	-0.33	0.04
Dizzy	650	1.69	0.84	1.06	0.47	0.03
Fatigue	650	2.05	1.11	0.76	-0.34	0.04

Forget	650	2.05	1.07	0.75	-0.28	0.04
Frustrated	650	1.87	0.99	1.01	0.34	0.04
Head	650	1.86	1.03	1.06	0.34	0.04
Hearing	650	1.53	0.80	1.43	1.35	0.03
Light	650	1.83	0.96	0.85	-0.28	0.04
Nausea	650	2.07	1.04	0.68	-0.30	0.04
Noise	650	1.40	0.73	1.93	3.75	0.03
Numb	650	1.58	0.84	1.50	2.05	0.03
Organize	650	1.58	0.86	1.40	1.19	0.03
Sad	650	1.94	1.04	0.87	-0.04	0.04
Sleep	650	2.43	1.14	0.42	-0.62	0.04
Taste	650	1.75	0.93	1.07	0.47	0.04
Tense	650	2.11	1.03	0.62	-0.33	0.04
Vision	650	1.39	0.74	1.92	3.05	0.03

Table S3

Edge Weight Estimates and Bootstrap Results for All Edges Included in the COVID- Network.

Edge	Weight	Mean	SD	LCI	UCI	P0
Annoyed--Frustrated	0.36	0.35	0.05	0.26	0.45	0.00
Appetite--Organize	0.16	0.12	0.10	-0.03	0.36	0.39
Balance--Clumsy	0.24	0.23	0.07	0.10	0.38	0.06
Clumsy--Light	0.18	0.14	0.10	-0.03	0.39	0.34
Concentrate--Forget	0.21	0.19	0.06	0.08	0.33	0.06
Decide--Annoyed	0.13	0.08	0.08	-0.03	0.29	0.49
Decide--Head	0.14	0.10	0.08	-0.03	0.31	0.37
Dizzy--Balance	0.32	0.31	0.05	0.22	0.43	0.00
Dizzy--Nausea	0.27	0.26	0.05	0.16	0.37	0.01
Dizzy--Vision	0.23	0.22	0.07	0.09	0.36	0.05
Forget--Decide	0.32	0.31	0.05	0.22	0.41	0.00
Forget--Head	0.25	0.24	0.05	0.15	0.35	0.01
Forget--Organize	0.13	0.12	0.08	-0.02	0.29	0.29
Head--Frustrated	0.14	0.10	0.08	-0.02	0.29	0.36
Head--Sleep	0.17	0.14	0.08	0.00	0.33	0.23
Hearing--Numb	0.28	0.27	0.06	0.15	0.41	0.02
Light--Hearing	0.25	0.24	0.06	0.13	0.37	0.03
Nausea--Vision	0.19	0.16	0.10	0.00	0.39	0.24
Noise--Numb	0.23	0.21	0.09	0.05	0.42	0.14
Organize--Frustrated	0.20	0.20	0.06	0.09	0.31	0.03
Organize--Head	0.18	0.16	0.08	0.02	0.34	0.16
Organize--Sad	0.17	0.15	0.08	0.01	0.33	0.18

Sad--Annoyed	0.13	0.09	0.08	-0.03	0.29	0.41
Sleep--Sad	0.14	0.09	0.08	-0.03	0.31	0.42
Taste--Appetite	0.17	0.11	0.10	-0.04	0.38	0.44
Tense--Frustrated	0.23	0.22	0.06	0.12	0.34	0.02
Tense--Sad	0.32	0.32	0.06	0.21	0.43	0.00

Note. LCI = Lower-bound of the confidence interval, UCI = Upper-bound of the confidence interval, P0 = proportion of the 1,000 sample bootstrap that did not contain a given edge.

Table S4

Edge Weight Estimates and Bootstrap Results for All Edges Included in the COVID+ Network.

Edge	Weight	Mean	SD	LCI	UCI	P0
Annoyed--Frustrated	0.31	0.30	0.04	0.22	0.40	0.00
Appetite--Concentrate	0.21	0.19	0.08	0.06	0.36	0.11
Balance--Clumsy	0.33	0.32	0.04	0.25	0.41	0.00
Clumsy--Decide	0.15	0.14	0.04	0.07	0.23	0.03
Clumsy--Organize	0.09	0.06	0.06	-0.04	0.21	0.52
Clumsy--Taste	0.13	0.09	0.08	-0.02	0.28	0.39
Concentrate--Fatigue	0.13	0.09	0.08	-0.03	0.30	0.45
Decide--Fatigue	0.11	0.08	0.06	-0.02	0.23	0.36
Decide--Head	0.23	0.23	0.05	0.14	0.33	0.00
Dizzy--Balance	0.47	0.46	0.04	0.39	0.54	0.00
Dizzy--Nausea	0.15	0.14	0.06	0.04	0.26	0.10
Dizzy--Vision	0.18	0.17	0.06	0.06	0.29	0.07
Fatigue--Tense	0.22	0.22	0.05	0.13	0.31	0.00
Forget--Decide	0.34	0.33	0.05	0.25	0.43	0.00
Forget--Head	0.28	0.28	0.04	0.19	0.36	0.00
Forget--Sleep	0.14	0.14	0.05	0.05	0.24	0.05
Head--Sleep	0.14	0.13	0.05	0.04	0.23	0.06
Hearing--Numb	0.31	0.31	0.04	0.23	0.40	0.00
Light--Hearing	0.28	0.28	0.04	0.20	0.37	0.00
Nausea--Hearing	0.15	0.14	0.06	0.03	0.28	0.13
Nausea--Sleep	0.12	0.08	0.07	-0.02	0.26	0.40
Noise--Numb	0.31	0.30	0.04	0.23	0.40	0.00
Numb--Forget	0.12	0.10	0.06	0.01	0.23	0.18
Organize--Frustrated	0.14	0.12	0.05	0.03	0.24	0.11
Organize--Head	0.41	0.40	0.05	0.32	0.50	0.00
Sad--Annoyed	0.15	0.14	0.05	0.05	0.26	0.07
Sad--Frustrated	0.30	0.30	0.04	0.21	0.38	0.00
Sleep--Tense	0.10	0.07	0.06	-0.03	0.22	0.40
Taste--Fatigue	0.13	0.09	0.08	-0.03	0.30	0.42

Tense--Frustrated	0.23	0.23	0.04	0.15	0.31	0.00
Tense--Sad	0.33	0.33	0.04	0.25	0.42	0.00
Vision--Concentrate	0.17	0.14	0.08	0.00	0.33	0.21
Vision--Hearing	0.20	0.19	0.06	0.09	0.31	0.04

Note. LCI = Lower-bound of the confidence interval, UCI = Upper-bound of the confidence interval, P0 = proportion of the 1,000-sample bootstrap that did not contain a given edge.

Table S5

Edge Weight Comparisons from the Network Comparison Test.

Edge	*p*-value
Annoyed--Frustrated	0.487
Appetite--Concentrate	0.145
Appetite--Organize	0.005
Balance--Clumsy	0.197
Clumsy--Decide	0.142
Clumsy--Light	0.037
Clumsy--Organize	0.499
Clumsy--Taste	0.191
Concentrate--Fatigue	0.088
Concentrate--Forget	0.022
Decide--Annoyed	0.250
Decide--Fatigue	0.769
Decide--Head	0.200
Dizzy--Balance	0.023
Dizzy--Nausea	0.064
Dizzy--Vision	0.432
Fatigue--Tense	0.025
Forget--Decide	0.758
Forget--Head	0.704
Forget--Organize	0.294
Forget--Sleep	0.210
Head--Frustrated	0.178
Head--Sleep	0.682
Hearing--Numb	0.621
Light--Hearing	0.608
Nausea--Hearing	0.500
Nausea--Sleep	0.210
Nausea--Vision	0.170
Noise--Numb	0.241

Numb--Forget	0.052
Organize--Frustrated	0.337
Organize--Head	< .001
Organize--Sad	0.024
Sad--Annoyed	0.715
Sad--Frustrated	0.002
Sleep--Sad	0.063
Sleep--Tense	0.147
Taste--Appetite	< .001
Taste--Fatigue	0.331
Tense--Frustrated	0.966
Tense--Sad	0.840
Vision--Concentrate	0.284
Vision--Hearing	0.108
